# Haute Couture or Ready-To-Wear? Tailored Pelvic Radiotherapy for Prostate Cancer Based on Individualized Sentinel Lymph Node Detection

**DOI:** 10.3390/cancers12040944

**Published:** 2020-04-10

**Authors:** Anne-Victoire Michaud, Benoit Samain, Ludovic Ferrer, Vincent Fleury, Melanie Dore, Mathilde Colombie, Claire Dupuy, Emmanuel Rio, Valentine Guimas, Thierry Rousseau, Maelle Le Thiec, Gregory Delpon, Caroline Rousseau, Stephane Supiot

**Affiliations:** 1 Nuclear Medicine Department, Institut de Cancérologie de l’Ouest, 44805 Nantes Saint-Herblain, France; annevictoire.michaud@chu-nantes.fr (A.-V.M.); vincent.Fleury@ico.unicancer.fr (V.F.); mathilde.colombie@ico.unicancer.fr (M.C.); maelle.lethiec@ico.unicancer.fr (M.L.T.); caroline.rousseau@ico.unicancer.fr (C.R.); 2 Radiation Oncology Department, Institut de Cancérologie de l’Ouest, 44805 Nantes Saint-Herblain, France; benoit.samain.lp@gmail.com (B.S.); melanie.dore@ico.unicancer.fr (M.D.); emmanuel.rio@ico.unicancer.fr (E.R.); valentine.guimas@ico.unicancer.fr (V.G.); 3 Medical Physics Department, Institut de Cancérologie de l’Ouest, 44805 Nantes Saint-Herblain, France; ludovic.Ferrer@ico.unicancer.fr (L.F.); claire.dupuy@ico.unicancer.fr (C.D.); gregory.delpon@ico.unicancer.fr (G.D.); 4 CRCINA CNRS Inserm, University of Nantes and Angers, F-44000 Nantes, France; 5 Urologic Clinic Nantes-Atlantis, 44800 Saint-Herblain, France; throuss@wanadoo.fr

**Keywords:** sentinel lymph node, radiotherapy, prostate cancer, SPECT/CT pelvic irradiation, personalized therapy

## Abstract

Prostate cancer (PCa) pelvic radiotherapy fields are defined by guidelines that do not consider individual variations in lymphatic drainage. We examined the feasibility of personalized sentinel lymph node (SLN)-based pelvic irradiation in PCa. Among a SLN study of 202 patients, we retrospectively selected 57 patients with a high risk of lymph node involvement. Each single SLN clinical target volume (CTV) was individually segmented and pelvic CTVs were contoured according to Radiation Therapy Oncology Group (RTOG) guidelines. We simulated a radiotherapy plan delivering 46 Gy and calculated the dose received by each SLN. Among a total of 332 abdominal SLNs, 305 pelvic SLNs (beyond the aortic bifurcation) were contoured (mean 5.4/patient). Based on standard guidelines, CTV missed 67 SLNs (22%), mostly at the common iliac level (40 SLNs). The mean distance between iliac vessels and the SLN was 11mm, and despite a 15mm margin around the iliac vessels, 9% of SLNs were not encompassed by the CTV. Moreover, 42 SLNs (63%) did not receive 95% of the prescribed dose. Despite a consensus on contouring guidelines, a significant proportion of SLNs were not included in the pelvic CTV and did not receive the prescribed dose. A tailored approach based on individual SLN detection would avoid underdosing pelvic lymph nodes that potentially contain tumor cells.

## 1. Introduction

Prostate cancer (PCa) is a very lymphatic malignancy [1] and cancer spread to lymph nodes is a strong unfavourable prognostic factor [2]. In localized PCa with a high risk of pelvic metastases, radiotherapy to the prostate and the pelvic lymph nodes combined with long-term hormone therapy was shown to improve overall survival and is therefore highly advocated [3]. However, the exact role of pelvic radiotherapy in PCa is still controversial. All studies in high-risk PCa patients included irradiation of the lymph nodes [3]. Whereas the GETUG-01 study did not show any survival advantage in irradiating pelvic lymph nodes based on the Roach formula [4,5], the RTOG 9413 study showed a progression-free survival benefit [6]. If studies could not definitely prove the utility of pelvic lymph node irradiation, this might be due to the fact that some micrometastatic lymph nodes are located outside the treated volume and therefore do not receive the full prescribed dose. Indeed, more pelvic relapses were found following conformal radiotherapy compared to the four-field technique, suggesting that a too small clinical target volume (CTV) may miss micrometastatic lymph nodes [7].

The definitions of pelvic lymph node volumes are highly variable [8]. The most widely used definition is the RTOG consensus that defines pelvic lymph node volumes based on pelvic blood vessels with a 7-mm expansion from the L5/S1 interspace to the obturator fossa [9]. However, this consensus is based on conventional CT scanning and does not take into account more recent data based on sentinel lymph node (SLN) lymphoscintigraphy (SPECT/CT) detection and lymph MRI [10,11]. Indeed, the prostate has multidirectional lymphatic drainage with significant inter-individual variability which makes it difficult to target all the relevant lymph nodes to be taken into account in a therapeutic strategy [12]. The SLN technique, which assumes that the pathologic status of the SLN reflects the status of other pathologic regional lymph nodes, appears relevant [13], to target the lymph nodes to be removed during surgical dissection or to be included in a radiation field for a radiotherapy treatment. The SLN is therefore most likely to contain metastatic cells. To support this, a surgical series demonstrated that a SLN biopsy has a strong sensitivity (95%) in detecting lymph node metastases [14]. In PCa the rate of false negative (FN) SLN in a review of the literature was approximately 2%, but varied widely among case series (0–23%). Therefore, before the SLN detection, it should be recommended to carry out imaging of pelvic nodes and exclude patients with enlarged nodes, in particular over 15 mm. Blockade of the lymphatic ducts with a tumor is the explanation for FN SLN [15]. When combined with extensive pelvic lymph node dissection, SLN detection can potentially reduce biochemical recurrence rates [16]. We showed that SLN detection modified the pelvic surgical dissection road map [13]. The problem of defining CTV and surgical pelvic dissection extension is the same: which lymph nodes should we target? We therefore hypothesized that SLN detection prior to radiotherapy might help to personalize patient care by altering the definition of pelvic radiotherapy volumes.

The aim of this study was to determine the impact of SLN SPECT/CT images in prostate cancer radiotherapy by describing the location of pelvic SLNs and calculating the dose received by each SLN located outside of the RTOG consensus pelvic volumes.

## 2. Results

### 2.1. Patient Population

From a previously published series [13], we selected 57 PCa patients with a risk of lymph node involvement greater than 15%. These were classified as intermediate (*n* = 37, 65%) and high-risk patients (*n* = 20, 35%) according to the D’Amico classification (Table 1). All patients were enrolled in a prospective study approved by Tours-Ouest 1 ethic committee on 18/12/2007 (2007-R-41; BRD07/11-M), and a signed informed consent was obtained.

### 2.2. Anatomical Distribution of SLN on Lymphoscintigraphy

**Figure 1:**
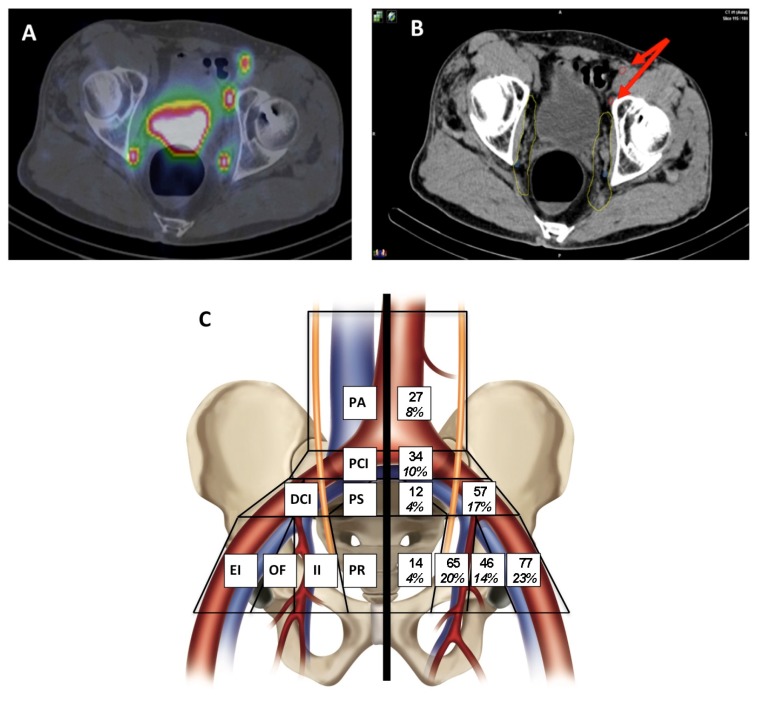
Anatomical distribution of sentinel lymph nodes (SLN) by lymphoscintigraphy (SPECT/CT) in different abdomino-pelvic regions. PA: para-aortic; PCI: proximal common iliac (between aortic bifurcation and L5-S1 interspace); DCI: distal common iliac (between L5-S1 interspace and iliac bifurcation); PS: pre-sacral; EI: external iliac; OF: obturator fossa; II: internal iliac; PR: peri-rectal. (**A**) Representative image of an axial lymphoscintigraphy (SPECT/CT); (**B**) two left- and right-sided II SLN are located within the RTOG clinical target volume (CTV) (yellow) and two left-sided EI SLNs are located outside the RTOGCTV N (red circles and red arrows); (**C**) distribution of sentinel lymph nodes.

We began by analyzing the anatomical distribution of SLNs by lymphoscintigraphy (SPECT/CT) as illustrated in Supplementary Figure S1. Pelvic lymph nodes are anatomically defined as being located beyond the aortic bifurcation. We detected 332 SLNs (mean 5.8, median 5 SLNs per patient) among which 27 were para-aortic and the remaining 305 pelvic (mean 5.4, median 5 SLNs per patient) (Figure 1). Among 15 of the 57 patients with para-aortic (PA) SLNs, 134 SLNs were segmented in total (average of 8.9 SLNs/patient). Among the 42 patients with no PA SLN, 198 SLNs were found (average of 4.7 SLNs/patient). The most common location of pelvic SLNs on SPECT/CT analysis was the common iliac (CI) area, followed by equivalent numbers in the internal iliac (II) and external iliac (EI) areas, and were least frequent in the obturator fossa (OF) and pre-sacral (PS) areas. 

### 2.3. Histological Distribution of Metastatic and Non Metastatic SLNs

**Figure 2:**
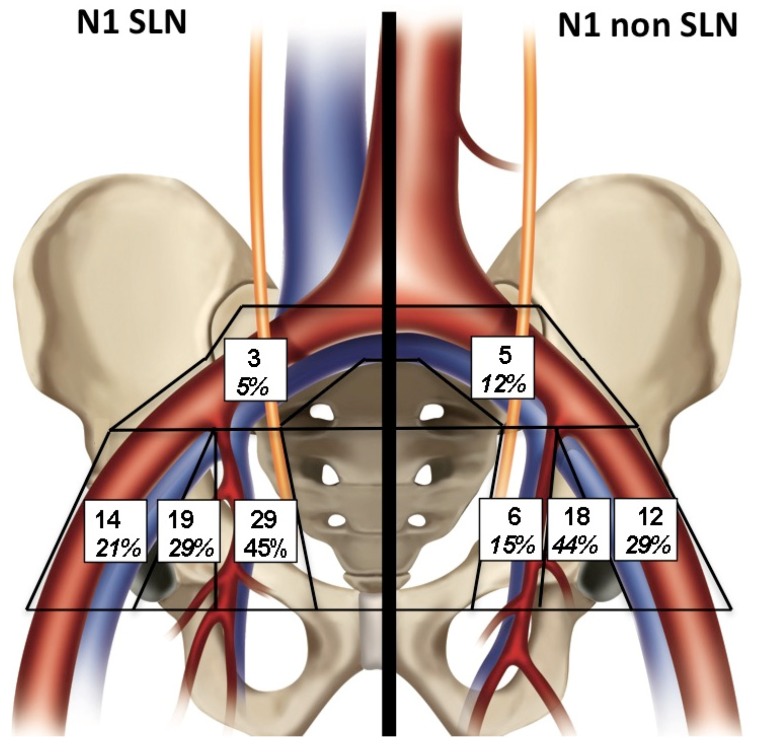
Distribution of metastasis-positive sentinel and non-sentinel lymph nodes by histological assessment.

Next we analyzed the histological distribution of metastatic and non-metastatic SLNs, because SLN SPECT/CT may miss large metastatic lymph nodes due to failed uptake of nanocolloid particles [17]. Since all 57 patients underwent extended surgical lymph node dissection, we could determine the anatomical location of 96 lymph node metastases located in the sentinel and non-sentinel lymph nodes (Table 2 and Figure 2). In 33 out of 57 patients (58%), at least one metastatic lymph node was found following pathology examination. Among these 33 patients, metastatic prostate cancer cells were found in the SLN only in 22 patients, and in both the SLN and non-SLN in the other 11 patients. A total of 65 SLNs were found to harbor metastatic prostate cancer cells. The majority (45%) were located in the II area and then in decreasing order, in the OF, EI and CI areas (Figure 2). A total of 41 non-SLNs were found to harbor metastatic prostate cancer cells. The location of metastasis-positive non-SLNs was different from the metastatic SLN distribution, and were predominantly located in the OF area (44%), then EI, II and CI areas. 

### 2.4. Pelvic Radiotherapy SLN Dose Distribution

**Figure 3:**
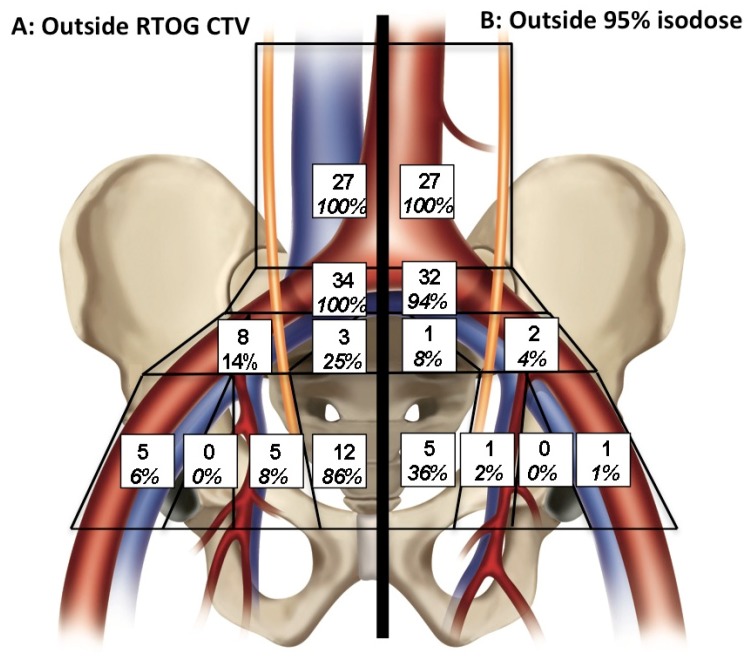
Pattern of distribution of SLNs in pelvic and para-aortic regions. Distribution of SLNs using RTOG-based CTV (**A**) and within the 95% isodose (**B**).

In our third analysis, we determined if a clinical target volume (CTV) contoured according to the RTOG contouring guidelines (RTOG CTV) could encompass all SLNs (excluding the PA SLN) in all 57 patients (Figure 3A). A total of 67 SLNs were detected outside the RTOG CTV in 32/57 (56.1%) patients (Table 3). For these patients, the mean SLN number outside the RTOG CTV was 1.18/patient (median 2/patient, range 0 to 6 SLNs per patient). By definition, none of the SLNs located within the PA and proximal common iliac (PCI) regions were included in the RTOG-based CTV. In the peri-rectal region, two SLNs out 14 were located within the other neighboring CTV volumes, but 12/14 were not identified by the RTOG CTV. Despite a rigorous definition, 25%, 14%, 8% and 6% of the PS, distal common iliac (DCI), II and EI SLN respectively, were not located within the RTOG CTV. 

In a fourth approach, we simulated pelvic radiotherapy fields and calculated the dose received by each SLN in the 33 patients for whom at least one SLN was found to be outside the CTV (Figure 3B). Indeed, the prescribed dose can be delivered to volumes located outside but close to the CTV because technical margins (PTV) and dosimetric fields may include neighboring SLNs. Since they were not included in the RTOG CTV, none of the PA SLN received the prescribed dose and only 6% and 36% of PCI and peri-rectal (PR) SLN received 95% of the prescribed dose, respectively. Between 1% to 4% of SLNs in the other areas did not receive 95% of the prescribed dose, with a minimal dose of 87% of the prescribed dose. In total, 26/32 patients had at least one SLN that did not receive the prescribed dose, and 63% of SLNs located outside the RTOG CTV would have not received 95% of the prescribed dose (median dose of 33% of the prescribed dose, range 5–93%). 

### 2.5. Distance between SLN and Regional Blood Vessels

**Figure 4:**
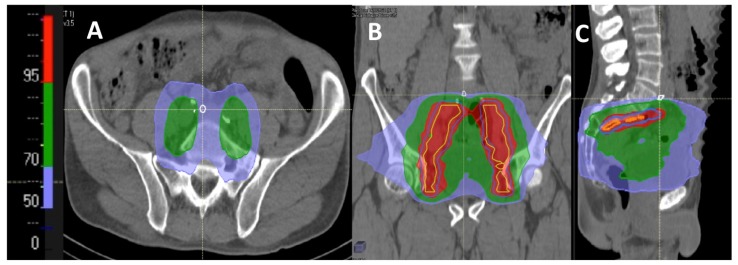
Example of dose distribution in axial (**A**), coronal (**B**) and sagittal (**C**) sections to a proximal common iliac SLN above the L5/S1 interspace located outside the RTOG CTV (white circle). Yellow: RTOG CTV; Red: 95% isodose, Green: 70% isodose; Blue: 50% isodose. The SLN appeared at the edge of the radiation field and would have received only a low dose (less than 40% of the prescribed dose).

In a fifth approach, we measured the minimal distance between pelvic blood vessels (veins and arteries) that was needed to encompass all surrounding SLNs (Table 4 and Figure 4). Whilst the RTOG consensus CTV is based on pelvic blood vessels (veins and arteries) with an expansion of 7 mm, SLNs might be located outside this volume. Of note, PS and OF areas are not defined by the nearest pelvic blood vessel and were therefore not analyzed. The RTOG consensus CTV does not include PA, PCI and PR areas. In the PA, PCI, DCI, EI and II areas, the mean distance of all SLNs from the nearest vessels was 8 mm (range 2–18 mm), 6 mm (range 2–18 mm), 8 mm (range, 2–20 mm), 11 mm (range 10–15 mm) and 14 mm (range 12–15 mm), respectively. In the DCI, EI and II areas, the mean distance of SLNs outside of the RTOG CTV from the nearest pelvic vessels was 13.5 mm, 15 mm and 10 mm, respectively. An expansion to 15 mm encompassed 100% of II and EI SLNs, but DCI SLNs were located up to 20 mm from the nearest common iliac vein or artery. The mean distance of PA SLNs from the nearest vessels was 10 mm for lateral (left or right) SLNs and 5 mm for SLNs located anteriorly or posteriorly of the aorta/vena cava inferior. 

## 3. Discussion

We hypothesized that SLN detection may help to personalize pelvic lymph node irradiation in prostate cancer patients. We analyzed SLN distribution and location in 57 intermediate and high-risk prostate cancer patients at high risk of pelvic involvement and simulated pelvic radiotherapy according to current guidelines. We found that SLNs are frequently located outside the boundaries that are usually recommended in consensus definitions, and that irradiation of a RTOG consensus-based CTV results in inadequate dosing to a significant proportion of SLNs. Given that prostate cancer is most likely to metastasize to the most proximal SLN and then spread to other areas, SLN SPECT/CT prior to radiotherapy would therefore help to personalize prophylactic pelvic radiotherapy in prostate cancer patients and avoid missing areas potentially harboring metastatic lymph nodes.

**Figure 5:**
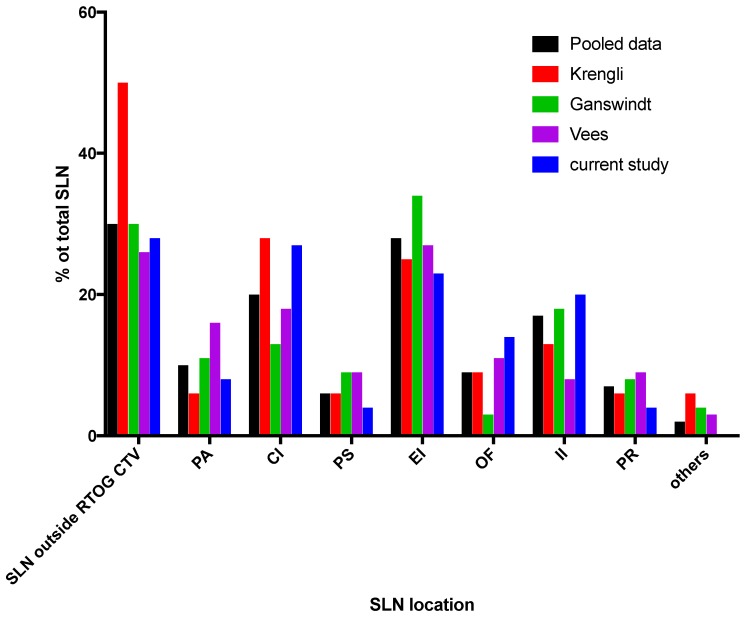
Pooled analysis of sentinel lymph node SPECT/CT in pelvic irradiation of prostate cancer. SLN location in different abdomino-pelvic areas.

The potential benefit of SLN SPECT/CT pelvic lymph node detection in prostate cancer has been assessed by a limited number of studies [10,18,19] (Table 5 and Figure 5). Most of these described the location of the SLNs and how pelvic CTVs should be modified. Our study analyzed a large number of SLNs in a homogenous series of patients. We identified a mean number of 5.8 SLNs per patient, which is similar to the other studies [19,20], but higher than Krengli et al. [10]. The distribution of SLN-positive areas was similar to the other studies, with a pooled analysis of all SPECT/CT data showing that EI and CI are the most common SLN areas with 28% and 20% of all SLN respectively, followed by II (17%), PA (10%), OF (9%), PR (7%), PS (6%) and others (2%). In our study, 28% of all SLNs were located outside the recommended consensus, and at least one SLN was missed in half of the patients. The number of missed lymph nodes is similar to other studies (between 26% and 38.5%), and these were most frequently located in the PA, PCI and PR areas which are not usually included in the RTOG consensus. Furthermore, other areas such as PS, CI, EI and II have up to 25% of the SLNs, and are not located within the RTOG CTV. It is of major importance to define the EI and II areas as precisely as possible because they harbor 29% and 15% of metastasis-positive non-SLNs, respectively, as determined by the pathology report following extensive lymph node dissection. In addition, our study showed that more than 60% of SLNs located outside the RTOG did not receive at least 95% of the prescribed dose (mainly lymph nodes above the L5/S1 interspace, but also in the PR area). This is similar to Krengli et al. where the dose to three of the four SLNs identified outside the RTOG CTV was inferior to 45 Gy [10]. Altogether, the four studies (including ours) show that in more than 50% of patients, 30% of all SLNs are not included in the boundaries of current pelvic CTV definition guidelines and do not receive the recommended irradiation dose, especially areas where SPECT/CT may miss true metastatic lymph nodes.

Should the RTOG guidelines be modified to include the proximal common iliac and para-aortic lymph nodes? There is no consensus as to the optimal superior border of pelvic radiotherapy fields. In our study, 34 SLNs outside the RTOG CTV were in the PCI area, and this represented the highest number and proportion of SLNs outside the RTOG CTV. Whilst the other SLN SPECT/CT studies did not separately evaluate the proximal and distal common iliac lymph nodes, they concluded similarly that the proportion of SLNs outside the RTOG CTV at the CI level varied from 27% to 63% of the cases [10,18,19,20]. These CI region SLNs were almost all located above the L5/S1 interspace (34 SLN/40), which is above the upper limit of pelvic CTV according to the RTOG recommendations [9]. Similar to the RTOG, others such as the UK CRUK PIVOTAL Group and GETUG [8] do not recommend that the superior border should encompass the entire common iliac nodal region. However, Spratt et al. [21] showed that more than 50% of patients treated for a localized prostate cancer by radiotherapy to the prostate only recurred at the CI level, and that only 42% of the first lymph node recurrences were covered by a classic pelvic radiotherapy field up to L5/S1. Furthermore, fluorocholine Positron Emission Tomography (PET)-based analysis of relapses following prostate-only radiotherapy showed that the upper field limit of pelvic radiotherapy could be extended to L2–L3 to cover 95% of lymph nodes at risk of relapse [22]. Our data support these findings and suggest that in patients at high risk of para-aortic metastases, prospective trials such as RTOG 0924 should determine if there is a role for extending the superior limit of the radiotherapy field [23]. 

Should the RTOG guidelines be modified to increase the margins around pelvic blood vessels? By defining the CTV according to the guidelines, which suggest a 7mm-margin around the regional blood vessels, 22% (excluding PA) or 28% (including PA) of the SLNs were located outside the RTOG CTV. According to our study, an enlargement of the pelvic CTV up to the aortic bifurcation with a margin around the CI vessels of 12 mm and a margin at the II level of 15 mm, while keeping the current recommendations on the other pelvic areas would cover 90.8% of the pelvic lymph nodes (277/305). Other teams have also defined pelvic volume in radiotherapy using other detection methods. Shih et al. [11] used MRI with ferumoxtran-10 injection. A new definition of pelvic CTV has been proposed with a 20 mm expansion around the iliac vessels allowing, in their study, to cover 94.5% of the pelvic lymph nodes. Using the same imaging modality for pelvic tumors (gynecology, prostate, bladder), Diniwell et al. [24] also proposed new margins to define the pelvic CTV N: 12 mm around the distal para-aortic vessels, 10 mm at the common iliac and internal iliac level, 9 mm at the external iliac level, a band 12 mm wide in front of the sacrum and an extension of 22 mm inside the pelvic wall. Hegemann et al. [25] came to the same conclusion using choline PET. Of the lymph nodes detected in 32 patients with essentially high-risk prostate cancer (81.25%) who were naive to any treatment, 43.6% were located outside the usual pelvic CTV. Almost half of them (44.1%) were at the para-aortic level and 14.7% at the common iliac level. More recently, Doughton et al. used intra-prostatic injection of Gallium-68 labeled nanocolloids for PET-CT detection of SLNs outside the usual drainage areas in an attempt to increase the detection sensitivity of the lymph nodes. The authors then showed that while the lymph node drainage pattern was similar to the usual techniques for detecting SLNs, it was faster, making it possible to detect aberrant non-lymphatic pathways, most notably venous [26]. Similarly, Prostate Specific Membrane Antigen (PSMA) PET showed that the common iliac nodes are a frequent site of both occult disease and recurrence following local treatment [27]. There is no recommendation for the definition of para-aortic CTV in prostate cancer patients. Based on our results, a 20 mm expansion around aorta/vena cava inferior encompassed all SLNs. However, we need to remove the small bowel, bladder, bony structures and muscles from the added margins, since there is no risk of involvement of lymphnodes in these structures. This implies, for example, that anterior margins should adjust to the vicinity of small bowel loops. In cervix cancer, contouring guidelines recommend defining para-aortic CTV as a 10-mm circumferential expansion (with 15-mm lateral expansion) from the aorta and 8-mm anteromedial and 6-mm posterolateral expansion from the inferior vena cava [28], but other authors recommend a contour that includes the vena cava, aorta, aorto-caval space, and the entire space to the left of the aorta laterally to the left psoas muscle [29]. 

Should we personalize the pelvic lymph node definition using SLN SPECT/ CT for each patient? One possibility would be to restrict pelvic CTV to areas where SLNs have been detected. However, restricting the CTV to the most frequently involved lymph node areas increases the risk of incomplete target coverage. Indeed, large metastatic lymph nodes may modify the lymphatic drainage and therefore may not be detected by SLN SPECT/CT [17]. Moreover, following extensive lymphadenectomy and histopathology we showed that metastatic cells could be detected in a large proportion of non-SLNs, therefore precluding targeting only the SLNs. A second possibility is to personalize pelvic CTV by enlarging the CTV to areas where SLNs are detected on SPECT/CT. This is of major importance for PS and PR lymph nodes, which were missed in a large proportion of patients (namely 25% and 86%, respectively) using RTOG contouring guidelines. Trying to irradiate all possible areas of lymphatic drainage in all patients may increase intestinal toxicity. Using IMRT, coverage of PR SLN is feasible without increasing the dose to the intestine [30]. Studies on a limited number of patients revealed the outcomes of SLN-based pelvic radiotherapy in prostate cancer [18,31]. SLN-based pelvic radiotherapy did not increase gastrointestinal or genitourinary acute toxicity [18]. In a series of 61 patients receiving pelvic SLN-based IMRT, biochemical relapse-free survival was high (73.8%) and the nodal clearance rate of SLN-IMRT reached 94% [31]. In view of the results of these studies, the question arises as to whether it is worthwhile performing SPECT/CT detection of pelvic SLNs before radiotherapy in all unfavorable intermediate or high-risk prostate cancer patients. Additional prospective clinical data are needed to validate this novel strategy and assess the safety of such treatment. 

This study has several limitations. Similar to other SLN SPECT/CT studies in prostate cancer, we did not evaluate the influence of a longer administration on the detection time of the nanocolloid prior to imaging. Similarly, patients with a high number of total SLN were more likely to present PA SLN, which may reflect individual differences of radiotracer uptake. Individual pharmacokinetic parameters and a longer time-interval between radiotracer injection and SPECT/CT may increase the number of identified SLN and modify the distribution of the SLN. Other imaging modalities may also redefine the pelvic CTV. For example, PSMA PET/CT imaging is another interesting approach to detect metastatic lymph nodes, but SLN SPECT/CT can detect smaller lymph nodes (2 mm vs. 5 mm) [32]. SLN SPECT/CT may also miss large metastatic lymph nodes, which would reduce the benefit of planning SLN SPECT/CT prior to radiotherapy of aggressive tumors [17].

## 4. Patients and Methods 

### 4.1. Population

This study is part of a previous study that determined the accuracy of the isotopic SLN technique in patients with intermediate or high risk localized PCa [13,33] for whom pelvic lymph node radiotherapy is debated. Among the 202 patients, we retrospectively selected patients with a risk of lymph node involvement higher than 15% according to the Roach formula [5]. The sentinel node lymphadenectomy took place at the same time as extended lymph node dissection in patients treated by radical prostatectomy or prior to external radiotherapy. All procedures performed were in accordance with the ethical standards of the institutional and national research committees and the 1964 Helsinki declaration and its later amendments or comparable ethical standards. The patients were informed of the use of their data within the framework of this research and we obtained their non-opposition.

### 4.2. Lymphoscintigraphy

The day before surgery, patients received two injections of 100 MBq of ^99m^Tc rhenium sulfide (Nanocis™, IBA, saclay, France) in a volume of 0.3 ml in each prostate lobe under endorectal ultrasound guidance. The mean injected activity of ^99m^Tc Nanocis^®^ was 248.5 ± 63.9 MBq. The radiochemical purity of the radiopharmaceutical was >95%. Antibiotic prophylaxis was administered to all patients one day prior to isotopic injection. A SPECT/CT acquisition centered on the abdominopelvic region was performed with a hybrid camera (SymbiaT2-Siemens, Erlangen, Germany) two hours after isotopic injection. This was a multi-slice spiral CT with the following characteristics: 130 kV, adapting the number of mAs depending on the patient’s morphology (CARE Dose 4D), 3 mm thick sections. The SPECT acquisition included 64 images of 30 s duration, each with a 256 × 256 matrix. SLNs were defined visually as hot spots on SPECT/CT images and fusion images were used to localize SLNs in relation to anatomic structures.

### 4.3. Target Volumes Definition

Each single SLN was individually segmented on SPECT/CT images using the threshold mostly used in the literature (41%) [34] using Dosisoft software. The RTOG consensus-based clinical target volumes (RTOG-CTV) were contoured on the SPECT/CT images using Iplannet (BrainLAB, Westchester, IL, USA) based on pelvic blood vessels (veins and arteries) with a radial expansion of 7 mm from which the small bowel, rectum, bladder and the bony and muscular reliefs were excluded. The proximal common iliac (PCI) CTV was contoured from the aortic bifurcation to the L5/S1 vertebral interspace. The distal common iliac (DCI) CTV was contoured from the L5/S1 interspace to the iliac bifurcation. The external iliac (EI) and internal iliac (II) CTVs were defined around the respective vein and artery and a 7 mm margin connecting the external and internal iliac contours was added to define the obturator fossa (OF) CTV. The pre-sacral (PS) CTV was defined as a 10 mm-thick band in front of the S1-S3 vertebrae. According to the RTOG consensus, RTOG CTV comprised DCI, EI, II, OF and PS CTVs [9]. A 5 mm margin defined the PTVs. To determine the distance between the SLNs and the pelvic blood vessels, the 3D radial expansion around pelvic veins and arteries was progressively increased until 100% of each SLN was covered by the CTV.

### 4.4. Dosimetric Study

For all patients with a SLN outside the RTOG CTV, we simulated a radiotherapy plan delivering 46 Gy in 23 fractions using a Raystation (Raysearch laboratories, Stockholm, Sweden). At least 95% of the dose had to be delivered to 95% of the total pelvic lymph node volume. Dose constraints to the bladder, rectum, small bowel and femoral heads followed the Quantec recommendations [35]. 

## 5. Conclusions

SLN mapping can help refine the definition of CTV for pelvic radiotherapy treatment in intermediate or high-risk prostate cancer patients. It would seem appropriate to increase the limits of pelvic CTV at least to the aortic bifurcation by observing a radial margin of 12 mm at the level of the CI proximal area, an internal margin of the II area of 15 mm, and moreover, to retain the current RTOG recommendations for the other pelvic lymph nodes. Personalizing the pelvic CTV by enlarging the CTV to areas where SLN are detected on SPECT/CT is feasible and needs to be evaluated in prospective trials.

## Figures and Tables

**Table 1:** Patient characteristics.

Characteristics	*n* = 57 (%)
Median age, years (range)	65 (51–78)
Tumor Stage	
T1	12 (21)
T2	30 (53)
T3	15 (26)
ISUP Grade	
Group 1	5 (9)
Group 2	15 (26)
Group 3	15 (26)
Group 4	14 (25)
Group 5	8 (14)
Percentage of positive biopsy cores (%) Mean, (median) Range	50.5 (44) 5.2–100
Initial PSA (ng/mL) Median, (range)	
<10	30 (53)
10 to 20	16 (28)
>20	11 (19)
D’Amico risk grouping	
Intermediate risk	20 (35)
High risk	37 (65)

**Table 2:** Distribution of metastasis-positive sentinel and non-sentinel lymph nodes by histological assessment.

Location	Number of Metastatic Sentinel Lymph Nodes (% of Total)	Number of Metastatic Non-Sentinel Lymph Nodes (% of Total)	Total
*CI*	3 (4.6)	5 (12.2)	8 (8.3)
*II*	29 (44.6)	6 (14.6)	35 (36.5)
*EI*	14 (21.5)	12 (29.3)	26 (27.1)
*OF*	19 (29.3)	18 (43.9)	37 (28.1)
Total	65 (100)	41 (100)	106 (100)

**Table 3:** Location of pelvic SLN within or outside the RTOG CTV.

Location	Total SLN Number (%)	SLN NUMBER Outside RTOG CTV (%)
CI (PCI and DCI)	89 (29.4)	40 (44.9)
II	65 (21.2)	5 (7.7)
EI	77 (25.2)	5 (6.5)
OF	46 (15.1)	0 (0.0)
PS	22 (7.1)	13 (59.1)
PR	6 (2.0)	4 (66.7)
Total	305	67 (22.0)

**Table 4:** Distance (mm) from the nearest blood vessels of SLNs located outside the RTOG CTV.

Region	Median Minimal Distance (mm)	Number of Lymph Nodes (%) Located within a CTV Based on an Expansion around Pelvic Blood Vessels			
		≤10 mm	≤12 mm	≤15 mm	≤20 mm
PA	7	16 (61)	19 (72)	22 (83)	27 (100)
PCI	6	30 (88)	31 (91)	33 (97)	34 (100)
DCI	13.5	1 (13)	4 (50)	6 (75)	8 (100)
II	15	0 (0)	2 (40)	5 (100)	5(100)
EI	10	3 (60)	4 (80)	5 (100)	5 (100)

**Table 5:** Summary and pooled analysis of sentinel lymph node SPECT/CT in pelvic irradiation of prostate cancer.

Study	Current Study		Krengli		Ganswindt		Vees		Total	
Number of patients	57		20		59		20		156	
Total number of SLN	332		32		324		104		792	
Mean number of SLN/patient	5.8		1.6		5.5		5.2		5.1	
SLN outside RTOG CTV	*n*	(%)	*n*	(%)	*n*	(%)	*n*	(%)	*n*	(%)
	94	(28)	16	(50)	98	(30)	27	(26)	235	(30)
Distribution of SLN										
PA	27	(8)	2	(6)	35	(11)	17	(16)	81	(10)
CI	91	(27)	9	(28)	41	(13)	19	(18)	160	(20)
PS	12	(4)	2	(6)	28	(9)	9	(9)	51	(6)
EI	77	(23)	8	(25)	111	(34)	28	(27)	224	(28)
OF	46	(14)	3	(9)	10	(3)	11	(11)	70	(9)
II	65	(20)	4	(13)	58	(18)	8	(8)	135	(17)
PR	14	(4)	2	(6)	27	(8)	9	(9)	52	(7)
Others	0	(0)	2	(6)	14	(4)	3	(3)	19	(2)

## Supplementary Materials

The following are available online at https://www.mdpi.com/2072-6694/12/4/944/s1, Figure S1: example of SPECT/CT in a patient with sentinel lymph nodes (red) within or outside the RTOG CTV (yellow) at different axial levels.
