# Supplementary material for: Haute Couture or Ready-To-Wear? Tailored Pelvic Radiotherapy for Prostate Cancer Based on Individualized Sentinel Lymph Node Detection
Source: Cancers (Basel). 2020 Apr 10;12(4):944. doi: 10.3390/cancers12040944 (PMC7226011; doi:10.3390/cancers12040944)
Supplement: Supplementary file 1 [file cancers-12-00944-s001.pdf]

cancers

Article

Haute Couture or Ready-to-Wear? Tailored Pelvic Radiotherapy for Prostate Cancer Based on Individualized Sentinel Lymph Node Detection

Anne-Victoire Michaud, Benoit Samain, Ludovic Ferrer, Vincent Fleury, Mélanie Doré, Mathilde Colombié, Claire Dupuy, Emmanuel Rio, Valentine Guimas, Thierry Rousseau, Maelle Le Thiec [1], Grégory Delpon, Caroline Rousseau and Stéphane Supiot

Supplementary Materials

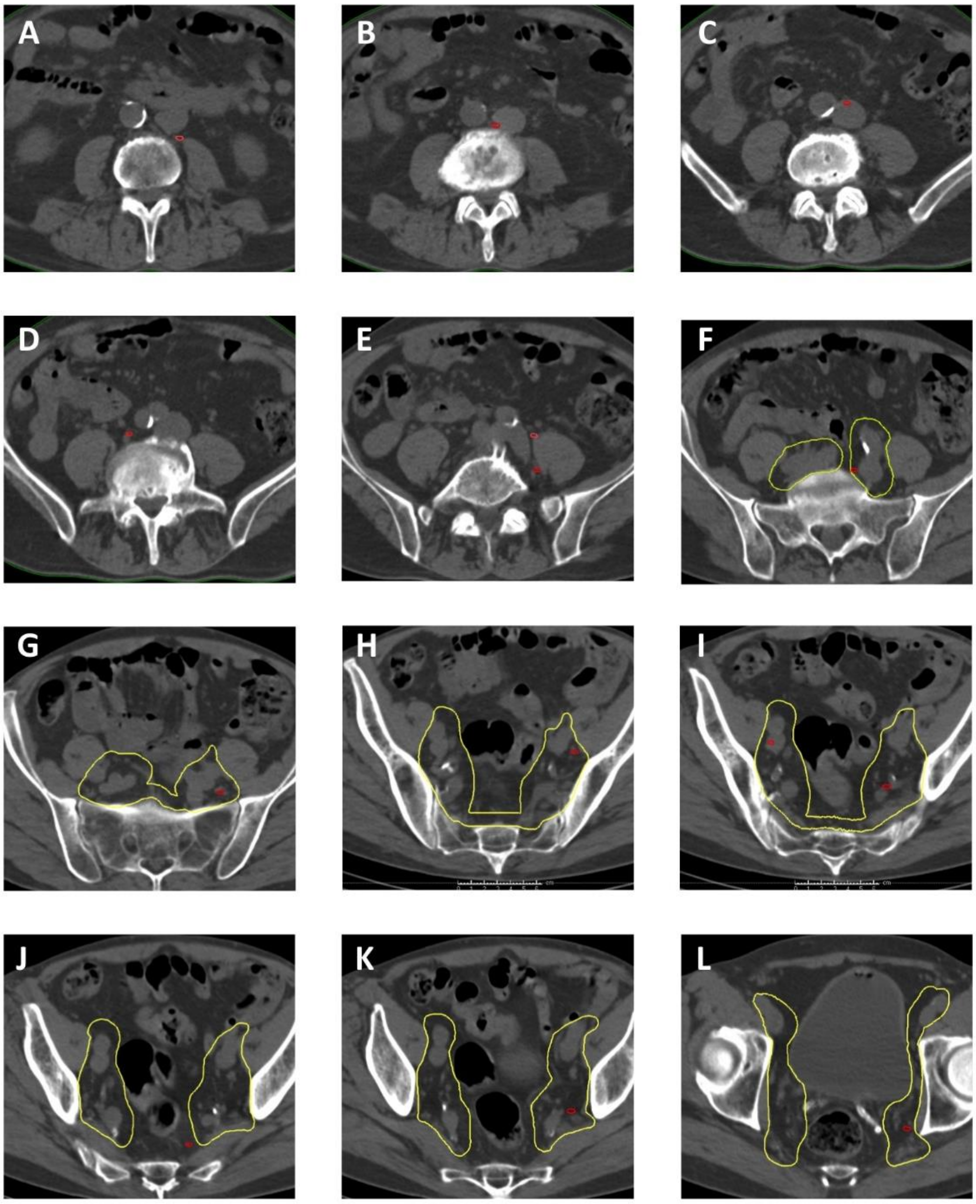

Figure S1. Example of SPECT/CT in a patient with sentinel lymph nodes (red) within or outside the RTOG CTV (yellow) at different axial levels. Para-Aortic (**A,B,C**) Proximal Common Iliac (**D,E**) Distal

Common Iliac (**F,G**) Internal and External Iliac (**H,I**) Presacral (**J**), Internal Iliac (**K**) Obturator Fossa (**L**) SLNs.

© 2020 by the authors. Licensee MDPI, Basel, Switzerland. This article is an open access article distributed under the terms and conditions of the Creative Commons Attribution (CC BY) license (http://creativecommons.org/licenses/by/4.0/).
